# Alexithymia and its impact on quality of life in a group of Brazilian women with migraine without aura

**DOI:** 10.1186/1129-2377-14-18

**Published:** 2013-02-25

**Authors:** Rebeca Veras de Andrade Vieira, Daniel Chaves Vieira, William Barbosa Gomes, Gustavo Gauer

**Affiliations:** 1 Federal University of Rio Grande do Sul, Porto Alegre, Brazil

**Keywords:** Migraine, Alexithymia, Self-reflection, Insight, Quality of life

## Abstract

**Background:**

Migraine is a type of primary headache widely known for its impact on quality of life of patients. Although the psychological aspects of the disease are receiving increasing attention in current research, some of them, as alexithymia, are still seldom explored. This study aimed to provide evidence on the relationships between markers of depression, anxiety, alexithymia, self-reflection, insight and quality of life in migraine.

**Methods:**

Forty female outpatients from a Brazilian specialized headache hospital service and a paired control group were compared.

**Results:**

The results revealed that women with migraine had higher levels of depression, anxiety and alexithymia, and lower levels of quality of life, self-reflection and insight, compared to controls. Quality of life in women with migraine was predicted by levels of depression and one alexithymia factor (ability to express emotions and fantasies). A binary regression analysis between clinical and control groups revealed the migraine group to comprise individuals with high anxiety, low quality of life in the physical domain and the presence of a concrete thinking style.

**Conclusions:**

The results highlight the relevance of considering psychological variables in the routine healthcare practices for migraine patients in general, while keeping steady attention to individual case features.

## Background

Migraine is a type of primary headache afflicting in average 11 percent of the global adult population and approximately 15.2 percent of the Brazilian adult population [1,2]. It is more prevalent in women than men, a pattern that can also be observed in the Brazilian population [2]. The relationship between migraine and psychological factors such as cognitive processing patterns (e.g., repetitive thinking and cognitive styles) and personality traits has received increasing attention within literature. Such research starts from the general premise that the way individuals perceive and express their ailment will have important effects in their relationship with treatment and in choosing strategies to cope with the disorder.

One of such relevant cognitive patterns is alexithymia, which is more common in migraine sufferers comparing to controls [3]. In a sample of medical students in Turkey, migraine was associated with alexithymic personality traits and comorbid psychiatric disorders [4]. There is also evidence of a positive association between alexithymia, depression and anxiety in migraine patients. Furthermore, when compared with episodic migraine patients, chronic migraine patients have significantly higher scores on measures of depression, but not alexithymia and anxiety [5]. In the same line, the relationship between alexithymia and migraine was probed in a migraine "repeaters" sample [6]. Repeaters show predominance of acute care for headache with high use and overuse of symptomatic medication [7]. The authors concluded that alexithymia and depressive mood associated with high disability may be a typical psychosocial pattern of "repeater" migraine patients.

The concept of alexithymia came about in the 1970’s, when Peter Sifneos observed peculiar characteristics in the emotional processing of psychosomatic patients [8]. Alexithymia is a multidimensional construct characterized by reduced ability to identify and describe feelings, limited capacity in engaging in fantasy or other imaginative activities, and externally-oriented thinking. It may be considered a deficit in one’s cognitive processing and regulation of emotional states [9,10].

Apart from and psychosomatic disorders, alexithymia has also been related to intensity of pain and functional impairment in populations with medical conditions such as neuromuscular disease, diabetes, hypertension and asthma [11-17]. As it interferes in the adaptive regulation of negative emotions resulting from stressing causes or psychological conflicts, alexithymia tends also to be stronger in individuals with chronic pain, such as rheumatoid arthritis, fibromyalgia, temporomandibular joint disorder and migraine [11,18]. However, there are important individual differences in alexithymia as in the intensity of pain, as much in the population in general as in patients with chronic pain. A positive correlation between alexithymia and the intensity of acute pain is found in healthy individuals, although in patients with chronic pain alexithymia it is not necessarily related to the gravity of pain, or it has been only related to the emotional, but not the sensorial dimension of pain [18].

The association between anxiety and alexithymia has also been documented [19]. On the one hand, some authors defend that alexithymia sufferers would be more susceptible to anxiety [20]. On the other hand, there is evidence suggesting that depression or anxiety might result in a reactive regression of the emotional development and then provoke alexithymic characteristics [21].

Several studies have also supported the association between alexithymia and depression, even when alexithymia measures appear to be relatively stable accross time when compared to depression measures, suggesting they might be different constructs [21-23]. However, mechanisms for the association between alexithymia and depression are not yet clear [24]. The investigation of psychiatric comorbidities in migraine has been highlighted in some studies not only because of its high impact on the individual’s quality of life but also due to the association between psychiatric comorbidities and suicide risk in those patients [25-27]. Moreover, the correct assessment of depression and anxiety in migraine patients seems to play an important role for developing an adequate preventive treatment strategy, preventing the evolution of migraine into a chronic illness [28].

There are empirical evidence in literature that alexithymia might be associated with a worse quality of life. But there is no consensus among authors if this association can be observed only in some dimensions, such as the psychological or the social quality of life, or if that happens in all of its dimensions [29,30]. Besides, some authors have found a negative correlation between satisfaction with life and alexithymia, even by taking into account confusion factors such as depression and physical health perception, which shows a possibility of low satisfaction with life being associated with difficulties in emotional processing, as is the case in alexithymia sufferers [31].

Alexithymia has been associated with other constructs related to the cognitive processing of psychic contents, such as thoughts, feelings and behaviors. For instance, self-reflection and insight were shown to be related to different psychological variables such as alexithymia, depression, anxiety and stress [32]. Self-reflection refers to the activity of inspecting and evaluating personal thoughts, sentiments and behaviors. Insight, on the other hand, concerns the state of internal understanding one has about one’s thoughts, sentiments and behaviors [33]. The self-reflection dimension has been positively correlated to anxiety and stress, but not to depression and alexithymia [32]. However, the factor insight was negatively correlated to depression, anxiety and alexithymia.

The literature still has not offered evidences about the impact of alexithymia on quality of life in migraine sufferers in the Brazilian context. There are few studies concerning possible correlations between alexithymia and depression, anxiety and other construct indicators involved in the self-monitoring of internal states such as self-reflection and insight. This study aimed at investigating the relations among the psychological factors alexithymia, self-reflection, insight, depression, anxiety; demographic variables; and aspects of the impacts of the disorder on these patients’ lives, such as severity of symptoms and quality of life in migraine sufferers as compared to a group of matched controls.

## Methods

### Participants

The clinical group was composed by 40 women with a migraine without aura diagnosis, according to the International Classification of Headache Disorders (2006) criteria. Participants’ age ranged from 21 to 59 years old (*M* = 43.58; *DP* = 10,71). The participants were selected among the outpatients registered at the Headache Unit of the Hospital de Clínicas de Porto Alegre (HCPA), a reference public hospital in city of Porto Alegre, state capital of Rio Grande do Sul, Brazil. The choice for evaluating exclusively women in this study is due to the profile of the population of outpatients attending the Hospital’s Headache Unit, comprising approximately 95% female.

The control group was formed by 33 women from the general population, who were receiving clinical care for other health issues than migraine, provided by general practitioners at the local public health center. Controls were paired with the clinical group by age, educational level and income status. Controls age ranged from 24 to 59 years old (*M* = 42.15; *DP* = 10,53). Some participants from the control group reported being in treatment for hypertension (7 cases), diabetes (2 cases), asthma (3 cases), labyrinthitis (1 case) and Ménièr’s Syndrome (1 case). The implications of that profile will be discussed in the last section of this article. At the public health care center, participants in the control group were questioned if they suffered of migraine or another type of headache, being excluded from the group those who presented any kind of headache, except infrequent episodic tensional headache. Participants in the clinical and control groups who met criteria for psychotic disorder or substance abuse, as screened by the instruments used in the study, were excluded.

### Instruments

#### CAGE

It is an instrument developed to track alcohol related disorders [34]. It consists of four short questions about alcohol ingestion followed by Yes or No answers. More than one affirmative response means the participant has alcohol related problems.

#### Self report questionnaire

SRQ is a psychiatric disorders screening questionnaire formed by 24 questions subdivided in two sections: 20 questions aim at “neurotic” disorders detection and the remaining four questions track “psychotic” disorders [35]. The “neurotic” disorders correspond to mood, anxiety and somatoform disorders, assessed by the SCID-IV-TR (Structured Clinical Interview for DSM-IV-TR) [36].

#### Brazilian version of the self-reflection and insight scale

The Self-Reflection and Insight Scale consists of a self-applied questionnaire formed by 20 items participants respond to a five-point Likert scale [32]. The results of the Brazilian adaptation study revealed satisfactory internal consistency indexes, with a Cronbach’s *alpha* of 0.902 for the Self-reflection dimension, and 0.825 for Insight [33].

#### Toronto alexithymia scale

It is a self-evaluation instrument formed by 26 items, and originally idealized to measure alexithymia degrees based on four factors [37]. The validation of the Brazilian TAS version with a clinical sample of 294 general hospital patients revealed a good internal consistency, with a Cronbach’s *alpha* of 0.72 [38]. The author performed a factor analysis of the scale and suggested a three factors model as more adequate for clinical samples. Items in the first factor (ALEX1) were associated with the ability to identify feelings and distinguish them from bodily sensations. Items in the second factor (ALEX2) relate to a concrete thinking style. Items in the third factor (ALEX3) concern the ability to express emotion and fantasy (daydreaming). With this release of three factors, the Brazilian version of TAS for clinical population is reduced to 22 items. There are currently no established cutoff points for the Brazilian population [38].

#### Beck anxiety inventory

It is a self-report scale that measures the intensity of anxiety-related signals and symptoms [39]. The instrument comprises 21 descriptive affirmatives of anxiety symptoms to be evaluated by the individual in a four-point scale that reflects the gravity of each symptom. The total score is obtained by adding up the scores of the individual items. The cutoff scores for the BAI are 0–10 (minimal), 11–19 (mild), 20–30 (moderate) and 31–63 (severe). Cronbach’s *alpha* for BAI in clinical samples varies from 0.74 to 0.88.

#### Beck depression inventory

It is a self-report scale with 21 items, each one with four alternatives, related to increasing degrees of depression gravity with scores from 0 to 3 [39]. The total score is the sum of the individual scores of the items. BDI cutoff scores are 1–11 (minimal depression), 12–19 (mild depression), 20–35 (moderate depression) and 36–63 (severe depression). Cronbach’s *alpha* in clinical-medical samples varies from 0.77 to 0.92.

#### WHOQOL-BREF

The short version of *World Health Organization's Quality of Life* (WHOQOL-100) assesses quality of life in the physical (WHO1), psychological (WHO2), social relations (WHO3) and environmental domains (WHO4) [40]. The instrument has shown satisfactory results on discriminant validity, criterion validity, concurrent validity, and test-retest and internal consistency reliability both for the specific domains and the complete scale (WHO).

### Procedure

Participants of the clinical group were found through the patients roll at the HCPA headache ambulatory between May and November, 2011. The instruments were applied in one occasion, on the same day of patients’ routine doctor’s appointment. Data collection was previously scheduled with the patients by phone and done at the Clinical Research Center. Participants in the control group were invited to participate in the study while they waited for their doctor’s appointment at the public health care center of their community. With their acceptance and after checking exclusion criteria, they were paired by age, educational level, and income to the participants of the clinical group. All the participants gave their informed consent prior to their inclusion in the study. The study received the approval by the Hospital's Institutional Review Board.

### Data analysis

The data matrix was inspected and the missing values were substituted by the average value of the case in the respective variable. Descriptive statistics were performed for the sociodemographic data for the total participants, as well as for the control and clinical groups in separate. Inferential statistics were also run for groups and overall sample (Chi-squared test, student’s t-test, Pearson correlation, analysis of covariance, multiple regression analysis, binary logistic regression), using SPSS (Statistical Package for Social Sciences), and adopting a 5% significance level.

## Results

Sociodemographic characteristics of the sample are shown at Table 1. Significant differences regarding those features between the control and clinical groups were not found. The same table features the values of duration of disease in months (DD), duration of treatment in months (DT), pain frequency in the last three months in days (PF) and an average grade attributed by the participants to their pain in the last three months in a scale ranging from 0–10 (PG) for the clinical group are described. To evaluate the grade attributed to the pain (PG) and the frequency of pain (FP) the authors used two questions from the Migraine Disability assessment Scale-MIDAS [41], a widely used measure of headache-related disability in migraine. The questions were "On how many days in the last 3 months did you have a headache?" and "On a scale of 0 to 10, on average how painful were these headaches? (where 0 = no pain at all and 10 = pain as bad as it can be".

**Table 1:** Sociodemographic characteristics of the sample

**Variables**	**Control group**	**Clinical group**	**Total**
Age: Mean (SD)	42,15 (10,53)	43,58 (10,71)	42,93 (10,58)
Range	24-59	21-59	21-59
Educational	Elementary = 14 (42,4)	Elementary = 20 (50)	Elementary = 34 (46,6)
Level: *f* (%)	High school = 11 (33,3)	High School = 12 (30)	High School = 23 (31,5)
	Professional = 3 (9,1)	Professional = 3 (7,5)	Professional = 6 (8,2)
	College = 5 (15,2)	College = 5 (12,5)	College = 10 (13,7)
Outcome (in current minimum wages): *f* (%)	Until 1 = 0 (0)	Until 1 = 2 (5)	Until 1 = 2 (2,7)
	From 1 to 3 = 22 (66,7)	From 1 to 3 = 24 (60)	From 1 to 3 = 46 (63)
	From 3 to 5 = 10 (30,3)	From 3 to 5 = 11 (27,5)	From 3 to 5 = 21 (28,8)
	From 5 to 10 = 1 (3)	From 5 to 10 = 3 (7,5)	From 5 to 10 = 4 (5,5)
Marital status: *f* (%)	Married = 14 (42,4)	Married = 19 (47,5)	Married = 33 (45,2)
	Divorced =5 (15,2)	Divorced = 6 (15)	Divorced = 11 (15,1)
	Single = 11 (33,3)	Single =13 (32,5)	Single = 24 (32,9)
	Widowed = 3 (9,1)	Widowed = 2 (5)	Widowed = 5 (6,8)
DD: M (SD)		242,10 (152,15)	
Range		36-564	
DT: M (SD)		57,52 (65,28)	
Range		3-312	
PF: M (SD)		27,65 (25,00)	
Range		0-90	
PG: M (SD)		8,03 (2,14)	
Range		0-10	

SD = standard deviation, *f* = frequency, % = percentage.

A comparison among the average levels of the variables investigated in the present study is shown at Table 2. Significant differences were observed in all variables between the control and clinical groups. The effect size of those differences ranged from *d* = 0.56 to 1.41, showing that the groups presented very different profiles regarding such variables.

**Table 2:** Dependent variables means in the clinical and control groups and group comparisons

**Variables**	**Mean (SD); Range**			
	**Control group**	**Clinical group**	**Total**	***t *value; Cohen's *d***
BDI	8,33(8,69);0-37	16,70(10,17);1-49	12,92(10,35) 0-49	*t* = −3,73 (71); p < 0,001; *d* = 0,88
BAI	6,18(7,46) 0-25	19,73(12,69) 0-51	13,60(12,57) 0-51	*t* = −5,67 (64,68); p < 0,001; *d* = 1,41
WHOQOL	95,79(12,35) 59-127	82,20(15,33) 50-112	88,34(15,54) 50-127	*t* = 4,19 (70,97); p < 0,001; *d* = 0,99
WHOQOL1	27,06(3,74) 16-35	21,39(4,65) 11-33	23,95(5,10) 11-35	*t* = 5,67 (71); p < 0,001; *d* = 1,34
WHOQOL2	22,73(3,25) 14-30	19,97(4,37) 9-28	21,22(4,12) 9-30	*t* = 3,08 (70,32); p = 0,003; *d* = 0,73
WHOQOL3	11,61(2,16) 8-15	9,90(2,38) 4-14	10,67(2,43) 4-15	*t* = 3,17(71); p = 0,002; *d* = 0,75
WHOQOL4	27,33(4,37) 16-28	24,73(4,89) 11-33	25,90(4,81) 11-38	*t* = 2,38 (71); p = 0,02; *d* = 0,56
ALEXITHYMIA	53,21(9,49) 32-70	63,72(8,30) 50-89	58,97(10,25) 32-89	*t* = −5,05 (71); p < 0,001; *d* = 1,20
ALEX1	26,30(7,70) 11-42	32,28(7,82) 15-50	29,58(8,27) 11-50	*t* = −3,27 (71); p = 0,002; *d* = 0,78
ALEX2	16,09(4,57) 9-30	19,30(5,46) 7-33	17,85(5,29) 7-33	*t* = −2,69 (71); p = 0,009; *d* = 0,64
ALEX3	10,82(2,20) 7-17	12,15(2,52) 5-17	11,55(2,45) 5-17	*t* = −2,38 (71); p = 0,02; *d* = 0,56
SR	32,64(7,24) 14-46	28,88(5,43) 13-40	30,58(6,55) 13-46	*t* = 2,53 (71); p = 0,01; *d* = 0,60
INS	21,39(5,75) 9-29	16,33(6,14) 5-29	18,62(6,45) 5-29	*t* = 3,61 (71); p = 0,001; *d* = 0,86

SD = standard deviation; SR: Self-reflection; INS: Insight.

Table 3 shows bivariate correlations between the investigated variables, also including the sociodemographic variables of the studies for the control and clinical groups. Table 4 features associations among the following variables: duration of disease (DD), duration of treatment (DT), pain frequency in the last three months in days (PF) and an average grade attributed by the participants to their pain in the last three months in a scale ranging from 0–10 (PG) with the other variables. Association between pain frequency (in days) in the last three months (PF), variables related to mental health (depression and anxiety) and quality of life (total and in the physical, psychological and environmental domains) can be observed. The duration of disease (DD) presented a positive association with the age of the participants, while the variable average score attributed by the participants to their pain in the last three months (PG) was positively associated to anxiety levels and negatively to insight capacity.

**Table 3:** Bivariate correlations between investigated variables

**Variável**	**Control group / Clinical group**^**a**^															
	**1**	**2**	**3**	**4**	**5**	**6**	**7**	**8**	**9**	**10**	**11**	**12**	**13**	**14**	**15**	**16**
1-BDI	-	0,61**	−0,76**	−0,67**	−0,77**	−0,52**	−0,64**	0,40*	0,40*	0,01	0,06	−0,23	−0,41**	0,19	−0,09	−0,08
2-BAI	0,62**	-	−0,49**	−0,51**	−0,45**	−0,11	−0,43**	0,22	0,41**	−0,23	−0,04	0,20	−0,54**	0,27	−0,15	−0,06
3-WHOQOL	−0,72**	−0,59**	-	0,91**	0,91**	0,69**	0,86**	−0,42**	−0,23	−0,18	−0,30	0,265	0,34*	−0,18	−0,11	0,02
4-WHOQOL1	−0,60**	−0,68**	0,83**	-	0,79**	0,53**	0,66**	−0,41**	−0,23	−0,19	−0,26	0,25	0,48**	−0,29	−0,05	−0,02
5-WHOQOL2	−0,58**	−0,31	0,87**	0,61**	-	0,53**	0,69**	−0,38*	−0,26	−0,15	−0,11	0,31*	0,37*	−0,09	−0,09	0,04
6-WHOQOL3	−0,50**	−0,30	0,68**	0,48**	0,73**	-	0,60**	−0,28	−0,05	−0,23	−0,27	0,16	0,00	−0,08	−0,10	−0,07
7-WHOQOL4	−0,57**	−0,44*	0,84**	0,56**	0,64**	0,41*	-	−0,36*	−0,155	−0,14	−0,38*	0,20	0,18	−0,06	−0,06	0,12
8-ALEXITHYMIA	0,41*	0,41*	−0,64**	−0,53**	−0,54**	−0,36*	−0,63**	-	0,70**	0,46**	0,11	−0,20	−0,58**	0,04	0,23	0,08
9-ALEX-1	0,60**	0,47**	−0,63**	−0,56**	−0,48**	−0,38*	−0,59**	0,80**	-	−0,24	−0,26	0,15	−0,64**	0,17	−0,01	0,12
10-ALEX2	−0,06	0,10	−0,22	−0,11	−0,28	−0,12	−0,21	0,61**	0,05	-	0,09	−0,49**	−0,03	−0,13	0,25	−0,12
11-ALEX3	−0,21	−0,09	−0,09	−0,07	−0,05	0,04	−0,18	0,26	−0,17	0,36*	-	−0,07	0,13	−0,12	0,25	0,13
12-SR	−0,31	−0,39	0,20	0,31	−0,04	−0,07	0,23	−0,34*	−0,28	−0,20	−0,11	-	−0,01	−0,11	0,00	0,21
13-INS	−0,72**	−0,56**	0,63**	0,57**	0,44*	0,52**	0,53**	−0,58**	−0,72**	−0,07	0,17	0,30	-	−0,20	0,11	0,00
14-Age	−0,17	−0,09	0,20	−0,02	0,21	0,11	0,43*	−0,21	−0,29	−0,03	0,19	0,20	0,09	-	−0,15	0,07
15-Education	−0,04	−0,09	0,05	0,01	0,01	−0,11	0,14	−0,05	−0,03	−0,01	−0,09	0,20	0,01	−0,08	-	0,41**
16-Outcome	0,04	0,14	−0,04	−0,07	−0,06	−0,19	0,13	−0,04	−0,15	0,19	−0,05	0,26	−0,03	0,02	0,65**	-

^a^Lower diagonal refers to the control group; upper diagonal represents the clinical group. *p <0,05; **p < 0,01.

**Table 4:** Correlations between specific variables in the clinical group

**Variables**	**DD**	**DT**	**PF**	**PG**
TL	0,21			
PF	−0,04	0,01		
PG	0,08	0,02	0,20	
BDI	0,18	0,24	0,45**	0,14
BAI	0,11	0,01	0,50**	0,37*
WHOQOL	−0,20	−0,19	−0,45**	0,00
WHOQOL1	−0,16	−0,04	−0,51**	−0,10
WHOQOL2	−0,11	−0,22	−0,43**	0,04
WHOQOL3	−0,13	−0,22	−0,21	0,23
WHOQOL4	−0,19	−0,22	−0,32*	−0,01
ALEXITHYMIA	0,08	−0,08	0,11	0,26
ALEX1	0,20	−0,02	−0,07	0,30
ALEX2	−0,15	−0,17	0,16	0,08
ALEX3	−0,02	0,02	0,24	−0,24
SR	−0,20	−0,13	−0,13	0,00
INS	−0,22	0,13	−0,25	−0,32*
AGE	0,42**	0,11	0,07	0,08
EDUCATION	−0,07	0,12	−0,09	−0,30
OUTCOME	−0,06	−0,08	−0,01	−0,14

*p < 0,05; **p < 0,01.

Considering the associations among variables described in Table 4, ANCOVAs were performed with the purpose of investigating differences between the control and clinical groups, controlling the co-variables effect. The co-variables considered in each model were those that presented significant associations with the dependent variable. Only the variables total alexithymia and anxiety kept differences between the groups after control of the shared variance with the other variables. So, the fact of belonging to the control or to the clinical groups explains, respectively, 10% and 12% of the variance of these variables.

Table 5 shows a multiple linear regression analysis identifying predictors of quality of life for each group and for the total sample. It also depicts those variables predicting pain frequency (in days) in the last three months (PF) for the clinical group. This analysis evidences that depression, anxiety and alexithymia have predictive power over quality of life in general and its domains for the total, control and clinical groups. Regarding specifically the clinical group, pain frequency (PF) was predicted by the variables anxiety and ALEX1.

**Table 5:** Linear regression models for quality of life and pain frequency (in days) in the last three months (PF)

**Sample**	**Variable**	Predictors*	***B***	***Beta***	**Adjusted**
Total	WHO	BDI	−1,13	−0,76	0,70
		Alex3	−1,35	−0,21	
		Alex2	−0,48	−0,16	
	WHO1	BDI	−0,22	−0,44	0,65
		BAI	−0,14	−0,36	
		Alex3	−0,39	−0,19	
		Alex2	−0,17	−0,18	
	WHO2	BDI	−0,28	−0,72	0,57
		Alex2	−0,15	−0,19	
	WHO3	BDI	−0,13	−0,56	0,37
		Alex2	−0,10	−0,21	
	WHO4	BDI	−0,29	−0,63	0,50
		Alex3	−0,61	−0,31	
Control	WHO	BDI	−1,04	−0,73	0,55
		Alex2	−0,71	−0,26	
		BAI	−0,27	−0,53	0,51
		Alex1	−0,15	−0,31	
	WHO2	BDI	−0,22	−0,59	0,39
		Alex2	−0,22	−0,31	
	WHO3	Ins	0,20	0,52	0,25
	WHO4	Alex1	−0,23	−0,41	0,49
		Alex3	−0,68	−0,34	
		BDI	−0,20	0,40	
Clinical	WHO	BDI	−1,12	−0,75	0,62
		Alex3	−1,54	−0,25	
	WHO1	BDI	−0,31	−0,67	0,44
	WHO2	BDI	−0,33	−0,77	0,58
	WHO3	BDI	−0,12	−0,52	0,25
	WHO4	BDI	−0,30	−0,62	0,50
		Alex3	−0,67	−0,34	
Clinical	PF	BAI	1,23	0,63	0,30
		Alex1	−1,04	−0,33	

* Predictors with a significance level ≤ 0.05 by stepwise estimation method.

Finally, a binary logistic regression analysis (depicted in Table 6) revealed that the variables that better distinguish the control and clinical groups are related to anxiety, the physical domain of quality of life (WHO1) and concrete thinking style, based on reality, without imagination and fantasy (ALEX2). The model reached a satisfactory level of correct classification of the cases, of 85%, proving adequate to predict belonging to the respective groups.

**Table 6:** Variables predicting pertinence to the clinical group

		**Binary logistic regression analysis**				
		Predictors*	**B (e.p.)**	***Wald *(gl)**	**Odds ratio**	**Correct classification 85%**
Total	Group	BAI	0,11	6,12	1,11	
		WHO1	−0,16	2,68	0,85	
		Alex2	0,17	4,96	1,18	

* Predictors with a significance level ≤ 0.05 by stepwise estimation method.

Omnibus = 5,81(1), *p* = 0,02; Hosmer-Lemeshow = 16,85(8), *p* = 0,03.

## Discussion

The lack of correlation between alexithymia and sociodemographic variables (age, income and educational level) goes against discoveries of other studies [42,43]. This might be due to the small size and relative homogeneity of the sample in this study for both groups. However, regarding the group of migraine sufferers, a lack of co-relation between alexithymia and sociodemographic variables was also found in a Turkish sample, which points towards a possible specificity in the grouping form of these variables in the migraine sufferers group [3].

Migraine sufferers have shown higher levels of depression, anxiety and alexithymia, and lower levels of quality of life, self-reflection and insight, compared to a group of matched controls. The statistical significance of those differences corroborates the empirical relevance of such variables in investigating migraine in what characterizes the diagnosed sufferers. Besides, showing high distinction for migraine comparing to the control group, the relevance is stressed by the fact that the same variables have not shown significant results, in terms of correlation, interaction or distinction between groups, with none of the sociodemographic data.

The scores for total alexithymia correlated negatively with self-reflection and insight for the total sample and the control group as well. However, in the clinical group the total score for alexithymia only correlated negatively with insight and not with self-reflection. This lack of correlation between alexithymia and self-reflection subscribes previous findings from a non-clinical sample [32].

Discussion of the current data requires resorting to conceptual matters. Alexithymia concerns incapacity or difficulty to express emotions through words. Insight refers to the individual capability of understanding one’s own thoughts, feelings and behaviors. Self-reflection is the individual practice of inspection and evaluation of one’s own thoughts, feelings and behaviors. Sensibility in perceiving internal states and the construct related to one’s understanding of these states (insight) are certainly associated with the capability of understanding and expressing feelings [32]. Insight, in particular, predicts several emotion and self-evaluation markers: individuals with higher insight have a better functioning on this kind of capability, and insight predicts well-being markers [44].

In the case of migraine sufferers of the present study, there might be cognitive information processing regarding internal states in some degree. However, since the correlation direction expected and found in the control group would be negative, it is possible that migraine disorder associated with a type of chronic pain that “seizes” the patient and is hard to manage because of its unpredictability makes sufferers effectively have a forced self-reflection practice. This self-reflection would be a perception of one’s internal states, such as paying attention to the pain during a crisis and its possible internal or environmental triggers.

A reason to evaluate self-reflection and insight in clinical groups is their potential application in contexts where attention and introspection abilities focusing on internal states are able to predict the success of interventions [44]. In the case of migraine, the abilities of this field might constitute either resources or limiting features of important aspects of pain and treatment management, such as observing crisis triggers and evaluating medication effects.

Pain frequency in the last three months (PF) was significantly correlated to depression, anxiety and total quality of life and in the physical, psychological and social domains. This result stresses the strength of approximation of these variables in migraine, even if it is not possible to draw casualty directions in this study because of its transversal character. The average grade attributed by participants to their pain in the last three months (PG) was negatively correlated to insight and positively to anxiety. Perception and subjective evaluation of pain might have been influenced in these individuals by their loss of insight capability. In this way, insight might be interpreted as a cognitive mediator between perception and expression of pain, with specific effect, because of its interaction with alexithymia in the case of migraines. Alexithymia is indeed reposted in literature as an increase factor for sensitivity to pain and more somatic complaints [45].

There remains the question of whether elevated somatic complaints, higher sensitivity and lower pain tolerance in correlation to alexithymia could generate a model or an operationalization of the mechanisms through which migraine-related information is processed. Perhaps alexithymia, as a phenomenon of having difficulty expressing feelings, is closer to an outcome function of a process starting with self-reflection as a perception of one’s internal states, continuing with insight as a quality of cognitive data processing, supporting the individual’s reasoning processes over causes and effects of their illness.

The logistic regression analysis indicated two alexithymia dimensions (ALEX2 and ALEX3) and depression as predictors of quality of life in the total sample. However, comparing the clinical and control groups, each one of those same alexithymia dimensions related to a specific predictor. In the migraine sufferers group, quality of life (WHO) had as predictor, with negative charge, the capability in expressing emotions and fantasies (ALEX3), and in the control group, a concrete thinking style, without imagination and fantasies (ALEX2). This result is interesting because it relates quality of life in migraine sufferers to the possibility of expressing emotion and fantasy, reinforcing the importance of developing spaces to express sickness and treatment representations.

In the clinical group, pain frequency in the last three months (PF) was predicted by the variables anxiety and ability in identifying and describing feelings and distinguishing them from bodily sensations (ALEX1). This might happen because these variables are related to one of the main triggers described in literature: emotional stress.

In the binary logistic regression analysis, the Hosmer-Lemeshow index suggested discrepancies between the observed and predicted classifications, meaning that 15 percent of the cases were classified wrongly by the model. That might have happened because the control group is formed by individuals recruited in the primary health care network, who frequently have other disorders (hypertension, diabetes and asthma) which are frequently associated with alexithymic traits [46-50]. However, this lack of “purity” in the control group along with the significant difference of averages in all variables (alexithymia, self-reflection, insight, depression, anxiety and quality of life) reinforces the distinction between migraine sufferers and non-sufferers and the importance of the assessment of those variables in migraine patients. On the other hand, by making an analysis inside the clinical group, one can perceive a broad variability in every one of the variables, which corroborates the idea of intragroup heterogeneity among migraine sufferers.

The binary regression analysis of the division variable of both clinical and control groups revealed that migraine might characterize individuals with high anxiety, low quality of life in the physical domain (WHO1), and presence of a concrete thinking style, without imagination and fantasies (ALEX2). These results indicate the importance of evaluating these features during the routine medical appointment and planning adequate therapeutics aiming to decrease anxiety and developing symbolization capacity in migraine sufferers.

## Conclusions

This study demonstrated that migraine sufferers presented higher levels of depression, anxiety and alexithymia, and lower levels of quality of life, self-reflection and insight, comparing to control groups. However seeming two completely different groups, there is a high variability in all variables studied inside each group, which shows an intragroup heterogeneity. Hence, the results reinforce that while such variables present themselves as relevant in migraine sufferers, health professionals must evaluate them in their daily practice, watching attentively every individual case.

## Competing interests

There are not conflicts of interests among the authors.

## Authors’ contributions

RVAV was in charge of the recruitment of patients, data collection, data analysis, and manuscript preparation. DCV contributed with the literature review and study design. GG contributed with the study design, data analysis, discussion of results and manuscript preparation. WBG contributed with study design and manuscript preparation. All authors read and approved the final manuscript.
